# Genomic Landscape of Head and Neck Squamous Cell Carcinoma Across Different Anatomic Sites in Chinese Population

**DOI:** 10.3389/fgene.2021.680699

**Published:** 2021-06-14

**Authors:** Yunhe Ju, Xingrao Wu, Huizhen Wang, Bin Li, Qing Long, Dadong Zhang, Hao Chen, Nianqing Xiao, Fugen Li, Shiwen Zhang, Shenggang Yang

**Affiliations:** ^1^Department of Radiotherapy, The Third Affiliated Hospital of Kunming Medical University, Kunming, China; ^2^3D Medicines Inc., Shanghai, China; ^3^Department of Head and Neck Surgery, The Third Affiliated Hospital of Kunming Medical University, Kunming, China

**Keywords:** HNSCC, targeted sequencing, genomic alteration, mutational landscape, Chinese population

## Abstract

**Background:**

The characteristics of head and neck squamous cell carcinoma (HNSCC) across different anatomic sites in the Chinese population have not been studied. To determine the genomic abnormalities underlying HNSCC across different anatomic sites, the alterations of selected cancer-related genes were evaluated.

**Methods:**

Genomic DNA samples obtained from formalin-fixed, paraffin-embedded tissues were analyzed using targeted sequencing in a panel of 383 cancer-related genes to determine the genomic alterations.

**Results:**

A total of 317 formalin-fixed, paraffin-embedded HNSCC specimens were collected, and a total of 2,156 protein-coding mutations, including 1,864 single nucleotide variants and 292 insertions and deletions, were identified across more than six different anatomic sites. Mutation loads were distinct across the anatomic sites. Larynx carcinoma was found with the highest mutation loads, whereas nasopharynx carcinoma showed the lowest mutation loads. A total of 1,110 gains and 775 losses were identified in the 317 specimens. Patients who had at least one clinically actionable alteration (levels 1–4 in OncoKB) were identified. One patient had an actionable alteration with level 1 evidence in OncoKB, *TEX10*-*NTRK2* fusion, who may benefit from larotrectinib or entrectinib treatment.

**Conclusion:**

The genomic profiling of HNSCC using targeted sequencing can identify rational therapeutic candidate genes suitable for the treatment of the HNSCCs.

## Introduction

Head and neck squamous cell carcinoma (HNSCC), the main type of malignancy in the head and neck, arises from the mucosal surfaces of the mouth, salivary glands, pharynx, larynx, nasal cavity, and paranasal sinuses, accounting for more than 90% of head and neck malignancies, and is the sixth most common malignant tumors in the world (Bray et al., 2018). The incidence of HNSCCs is relatively low in China: according to GLOBOCAN 2012, the estimated age-standardized incidence rate in China is 2.7 per 100,000 compared with the world rate of 8.0 per 100,000 (Li et al., 2015). However, the total case number is largely due to the large population base. There were reportedly ∼75,000 new cases in 2015, of which 37,000 died from HNSCC (Chen et al., 2016).

Phenotypic, etiological, biological, and clinical heterogeneities are characteristics of HNSCC. Alcohol and tobacco consumption, human papillomavirus, particularly human papillomavirus type 16 infection, and Epstein–Barr virus infection are the risk factors for different types of HNSCCs derived from various anatomic sites (Niedobitek, 2000; Hashibe et al., 2006; Boffetta et al., 2008; Gandini et al., 2008; Chaturvedi et al., 2011; Young and Dawson, 2014; Yoshizaki et al., 2018). HNSCCs are well recognized as a particularly challenging class of tumors to treat. Despite surgery, radiation, and chemotherapy, such treatments for HNSCC can result in cosmetic deformity and functional impairment of vital functions, and >50% of HNSCC patients die from this disease (Bray et al., 2018) with a 5-year survival rate of only approximately 50% (Leemans et al., 2011). Despite the approval of cetuximab (Vermorken et al., 2007, 2008), a monoclonal antibody against epidermal growth factor receptor, the survival rates of HNSCC have improved very little over the past 40 years (Stransky et al., 2011; Du et al., 2019). Low survival outcomes in combination with significant toxicity of current treatment strategies emphasize the necessity for novel therapeutic modalities.

Similar to all solid tumors, HNSCC is thought to be initiated and progress through a series of genetic alterations. Comprehensive molecular profiling leads to the development of “personalized” or “precision” medicine. By promoting molecular diagnosis and targeted therapies, treatment of certain HNSCCs may soon be fundamentally transformed. Several studies have characterized alterations in a single anatomic site of head and neck in the Chinese population, including oral squamous cell carcinoma (Song et al., 2014; Izumchenko et al., 2015; Ma et al., 2018), nasopharyngeal carcinoma (NPC) (Zhang et al., 2017; Tu et al., 2018), and laryngeal squamous cell carcinoma (Zhang et al., 2014; Tao et al., 2018). These studies characterized the genomic alterations in these carcinomas. However, few studies describe and compare the characteristics of HNSCCs across different anatomic sites in the Chinese population.

To gain a comprehensive view of the genetic alterations underlying HNSCCs across different anatomic sites, we performed targeted deep sequencing on 317 samples from several anatomic sites in Chinese HNSCC patients. The genomic alterations of these HNSCCs were analyzed and compared.

## Methods

### Clinical Cancer Specimens

The HNSCC samples gathered for this study were approved by the Ethics Committee of the Third Affiliated Hospital of Kunming Medical University (No. QT201918; Kunming, Yunnan, China). Two pathologists reviewed a total of 317 formalin-fixed, paraffin-embedded (FFPE) tissues to make sure cancer cell contents were ≥20% before DNA extraction. The HNSCC samples were collected from September 2017 to March 2020 and sequenced in the Research and Development Institute of Precision Medicine, 3D Medicine Inc. (Shanghai, China). All patients provided written informed consent for their samples to be examined and their clinical data to be utilized.

### Targeted Sequencing

For each clinical FFPE HNSCC specimen, genomic DNA was extracted using QIAamp DNA FFPE Tissue Kit (Qiagen) and then quantified by PicoGreen fluorescence assay (Invitrogen). Fifty to 200 ng of DNAs were fragmented to around ∼200 bp by sonication (Covaris) and constructed into sequencing libraries with KAPA Hyper Prep Kit (Kapa Biosystems) according to the protocol. Target regions covering 383 cancer-related genes were captured with baits for each library. The captured libraries were then amplified with polymerase chain reaction, followed by purification with 1.8 × SPRI, quantification by Qubit 3.0 (Life Technologies). Finally, libraries were sequenced on an Illumina Nextseq 500 platform.

### DNA Alterations Analysis

#### Sequencing Data Processing

Raw sequencing reads were mapped to the human reference genome (hg19) using BWA-MEM v0.7.12 (Li and Durbin, 2010) with default settings. Mapped reads were then sorted and converted to BAM format using Picard (version 2.0.1)^1^, followed by the PCR duplicate removal process. For each BAM file, sequencing metric collections were summarized using an in-house script.

#### Base Substitution and Small INDEL Calling

A Bayesian methodology-based self-developed algorithm validated previously (Su et al., 2017) was used for base substitutions and small INDEL calling. A series of filtering models were applied to remove artifactual mutations in the calling algorithm, including corrections for background error, strand bias, base quality, mapping quality, and short tandem repeat regions. Somatic variant calls were then chosen with annotated mutations based on the following criteria: (1) for a given site, reads supported the alternative allele ≥ 8 and variant allele frequency ≥ 0.1; (2) only exonic or splicing sites were selected; (3) sites with strand bias ≥ 0.9 were removed; (4) INDELs longer than 40 bp were removed; and (5) sites with allele frequency larger than 0.015 in either of 1000 Genome Project or ESP6500 database were removed.

#### Copy Number Alteration Calling

A self-developed algorithm (in publishing) was used for the detection of copy number alterations. Briefly, we firstly built a mixed panel-of-normal (PON) using several normal samples. For each sample, including tumor and mixed PON, sequencing coverage depth was calculated by a fixed bin size across the targeted regions and normalized for GC content. A log2 ratio profile was obtained by dividing the normalized depth of each tumor sample by the mixed PON for all bins. A circular binary segmentation procedure was performed on the log2 ratio values to obtain copy number segments. To estimated tumor ploidy and purity, we used the B-allele frequency information of ∼5,000 selected single-nucleotide polymorphism loci from the human genome. We calculated the absolute copy number for each segment based on the log2 ratio values, tumor ploidy, and tumor purity.

### DNA Rearrangement Analysis

Genomic rearrangements were identified using a self-developed algorithm (in publishing). Briefly, the tag information was extracted from BAM files from BWA-MEM for the clipped reads. Then, reads mapped to separate chromosomes or at a distance of more than 2 kb were selected for the detection of genomic rearrangements. The rearrangement results contained translocation, inversion, long deletion, etc.

### Identification of Microsatellite Instability

To detect microsatellite instability (MSI) for each sample, 100 MS loci (repeat region from the human genome) were added into our targeted panel, and probes were specially designed for these loci. For each microsatellite locus, the distribution differences of repeat lengths were compared between tumor and paired normal samples. Each locus was classified as an MSI-high (MSI-H) or MSI-stable site, whether the differences were statistically significant or not. A sample was classified as MSI-H if there were not less than 75 MSI-H loci in the sample, otherwise MSI-stable.

### Statistical Analysis

Wilcoxon rank-sum test was used to compare the tumor mutation load of HNSCCs across different anatomic sites. The comparison of alteration frequencies across anatomic sites was conducted using a proportion test with continuity correction. The differences in alteration frequency between this study and The Cancer Genome Atlas (TCGA) dataset were established using a proportion test with continuity correction. All of the statistical analyses were performed with R.

## Results

### Clinical Specimens and Targeted Sequencing of Head and Neck Squamous Cell Carcinoma

In this study, a total of 317 HNSCC patients (Supplementary Table 1) were enrolled. FFPE samples and matched normal controls (peripheral white blood) were collected and subjected to targeted sequencing in a panel of 383 cancer-related genes (Supplementary Table 2) using Illumina Nextseq 500. The mean coverage of the sequencing depth was 542 × across the capture regions for all samples, whereas it was 730 × and 343 × for the examined tumor samples and the matched normal controls, respectively. According to the anatomic sites, the present cohort consisted of patients with paranasal sinus carcinoma (*n* = 4), NPC (*n* = 83), larynx carcinoma (LC, *n* = 60), oral cavity carcinoma (OCC, *n* = 111), hypopharynx carcinoma (HPC, *n* = 36), oropharynx carcinoma (*n* = 7), and 16 with unknown anatomic sites.

### Characterizing the Somatic Single-Nucleotide Variants and INDELs

**FIGURE 1:**
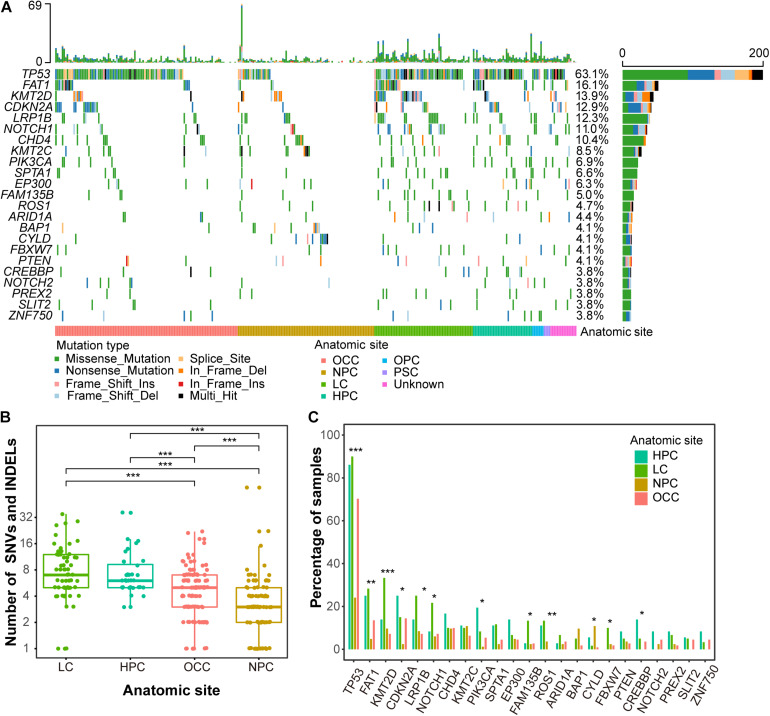
Mutation landscape of Chinese HNSCCs across different anatomic sites. **(A)** Waterfall plot of top 23 frequently mutated genes. Each row indicates a gene, and each column represents a patient. Patients are sorted by anatomic site. Bars on left show number of samples mutated for genes, whereas bars on top represent number of mutation for samples. Different mutation types are represented by different colors. **(B)** Mutation load by anatomic sites. X-axis indicates anatomic sites, and y-axis indicates number of mutations, including SNVs and INDELs. Each point represents one patient. **(C)** Mutation frequency of recurrent genes by anatomic sites. Anatomic sites are shown in different colors. *p < 0.05, **p < 0.01, ***p < 0.001.

The most common mutations in HNSCC were C > T transitions (42.2%), followed was C > A transversions (22.4%), and the distribution of mutation spectrum in our cohort was similar to that in TCGA HNSCC cohort (Cancer Genome Atlas Network (CGAN), 2015) (proportion test *P* = 0.95; Supplementary Figure 1). A total of 2,156 candidate somatic mutations [including 1,864 single-nucleotide variants (SNVs) and 292 INDELs involved in 273 genes] were identified in 317 samples with a median of five mutations (range 0–84). A mean of 5.5 non-synonymous mutations (including both SNVs and INDELs) per tumor was identified, resulting in a mean mutation rate of 4.2 mutations/Mb. This mutation rate was comparable with a previously published oral squamous cell carcinoma study (4.5 mutations/Mb) using a panel of 409 cancer-related genes (Nakagaki et al., 2017) and was more than twice as high as the rate seen in other HNSCC studies (Stransky et al., 2011; India Project Team of the International Cancer Genome Consortium (IPTICGC)., 2013; Vogelstein et al., 2013; Nakagaki et al., 2017). The results suggest that the cancer-related gene panel can successfully detect somatic mutations in FFPE samples. Of the top 23 highly mutated genes (mutated in ≥ 12 tumors), *TP53* was the most frequently mutated gene (63.1%), followed by *FAT1* (16.1%), *KMT2D* (13.9%), *CDKN2A* (12.9%), *LRP1B* (12.3%), *NOTCH1* (11.0%), and *CHD4* (10.4%) (Figure 1A). The mutation frequencies of recurrent genes were highly consistent with those in the TCGA dataset (Cancer Genome Atlas Network (CGAN), 2015) (*R*^2^ = 0.93; Supplementary Figure 2). However, four genes were significantly different. *PIK3CA* [6.9 vs. 18.4%; false discovery rate (FDR) = 1.7e-04, proportion test] and *CDKN2A* (12.9 vs. 21.9%; FDR = 0.011, proportion test) were observed with lower mutation frequency in the current Chinese cohort, whereas *CHD4* (10.4 vs. 2.9%; FDR = 2.0e-04, proportion test) and *BAP1* (4.1 vs. 0.6%; FDR = 0.0079, proportion test) were found with a higher mutation frequency in the current Chinese cohort. The mutational frequencies of recurrently mutated genes were also consistent between the NPC subgroup in the current Chinese cohort and NPC cohort from National University Hospital Singapore (Lin et al., 2014), and no significant statistical differences were found across the compared top mutated genes between the two datasets (Supplementary Figure 3).

The overall mutational loads across different anatomic sites of HNSCC were distinct. Due to the limited sample numbers, oropharynx carcinoma (*n* = 7) and paranasal sinus carcinoma (*n* = 4) were excluded from this analysis. LC had the highest mutation rate with a mean of 8.3 non-synonymous mutations per tumor, whereas NPC showed the lowest mutation rat with a mean of 3.6 non-synonymous mutations (Figure 1B and Supplementary Table 3). The mutational loads of OCC (an average of 4.5 non-synonymous mutations per tumor) were lower than LC and HPC (7.3 non-synonymous mutations per tumor) but higher than NPC. The lowest somatic mutational loads of NPC are consistent with previous studies (Lin et al., 2014; Zheng et al., 2016; Li et al., 2017).

The mutational frequencies of highly mutated genes were also different across different anatomic sites. *TP53* had the highest discrepancy across HPC, LC, NPC, and OCC, with mutation frequencies of 86.1, 90.0, 24.1, and 70.3% (FDR = 1.2e-16, proportion test) (Figure 1C). The mutational frequency of *KMT2D* was higher in LC (33.3%) than in HPC (13.9%), NPC (9.6%), and OCC (7.2%) (FDR = 2.9e-04, proportion test). Most genes presented with a lower mutation frequency in NPC than in the other anatomic sites except *CYLD* and *BAP1*, which were highly mutated in NPC (Figure 1C). The mutation frequency of *CYLD* in NPC patients was 10.8% (10 mutations in nine samples, 9/83), whereas that in the other three anatomic sites was only 1.9% (4/207). Most *CYLD* mutation types in NPC were truncating mutations that were caused by mutation at splice site (1 of 10, 1/10), frameshift insertion (1 of 10, 1/10), and a non-sense mutation (6 of 10, 1/10) (Supplementary Figure 4). *BAP1* was mutated in 9.6% (8/83) of NPC patients, whereas it was mutated in only 2.4% cases (5/207) in the other three anatomic sites. Interestingly, *CYLD* was mutually exclusive with *TP53* and *BAP1* in NPC patients (Supplementary Figure 5).

### Characterizing of Somatic Copy Number Variations

**FIGURE 2:**
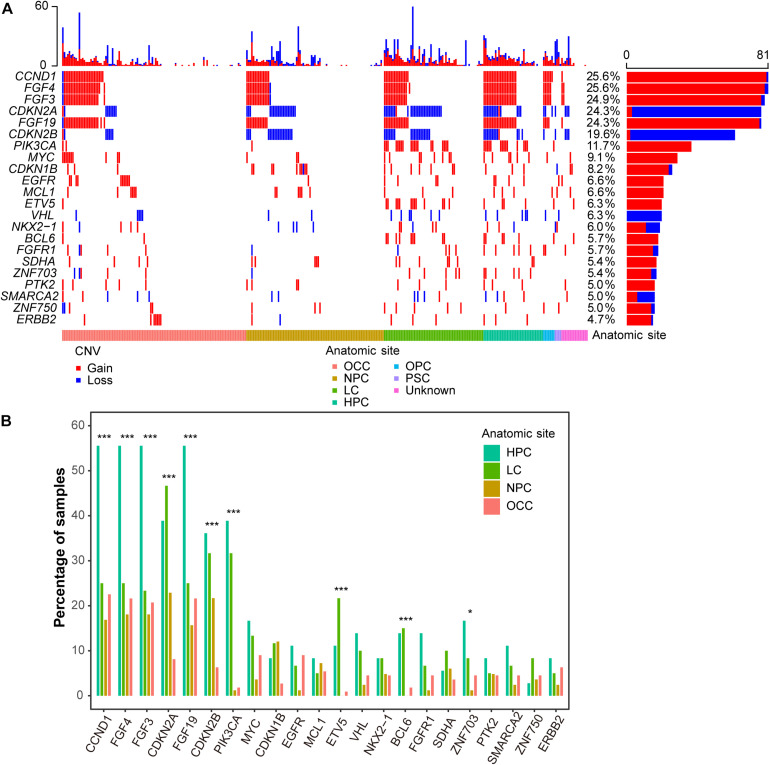
CNV landscape of Chinese HNSCCs across different anatomic sites. **(A)** Waterfall plot of top 22 frequent CNV genes. Each row indicates a gene, and each column represents a patient. Patients are sorted by anatomic site. Bars on left show number of samples with CNV in genes, whereas bars on top represent number of CNV for samples. Copy number gain and loss are represented in red and blue. **(B)** CNV frequency of recurrent genes by anatomic sites. Anatomic sites are shown in different colors. *p < 0.05, ***p < 0.001.

Copy number variations (CNVs) duplicated or deleted in the segments of the genome were also analyzed. A number of candidate genes were identified as recurrent somatic CNVs (Figure 2A). In total, 1,885 CNVs [1,110 (58.9%) gains and 775 (41.1%) losses] were identified in all 317 samples, resulting in a median of three CNVs per tumor (range 0–60). *CCND1* was the most frequently altered gene mainly with copy number gain, occurring in 25.6% (81/317) samples, including 80 copy number gains and 1 copy number loss, followed by *FGF4* (25.6%), *FGF3* (24.9%), and *FGF19* (24.3%). Other frequently altered genes with copy number gain were *PIK3CA* (11.7%), *MYC* (9.1%), and *CDKN1B* (8.2%). The most commonly altered gene, mainly with copy number loss was *CDKN2A*, occurring in 24.3% (77/317) of cases, including 3 gains and 74 losses, followed by *CDKN2B* (19.6%), including 2 gains and 60 losses (Figure 2A).

Interestingly, the CNV frequencies of recurrent genes across different anatomic sites were also different. Generally, most of the genes in HPC had the highest CNV frequencies (Figure 2B). *PIK3CA* had the highest discrepancy across HPC, LC, NPC, and OCC, with CNV frequencies of 38.9, 31.7, 1.2, and 1.8% (FDR = 1.6e-11, proportion test), respectively.

### Identification of Fusions

**FIGURE 3:**
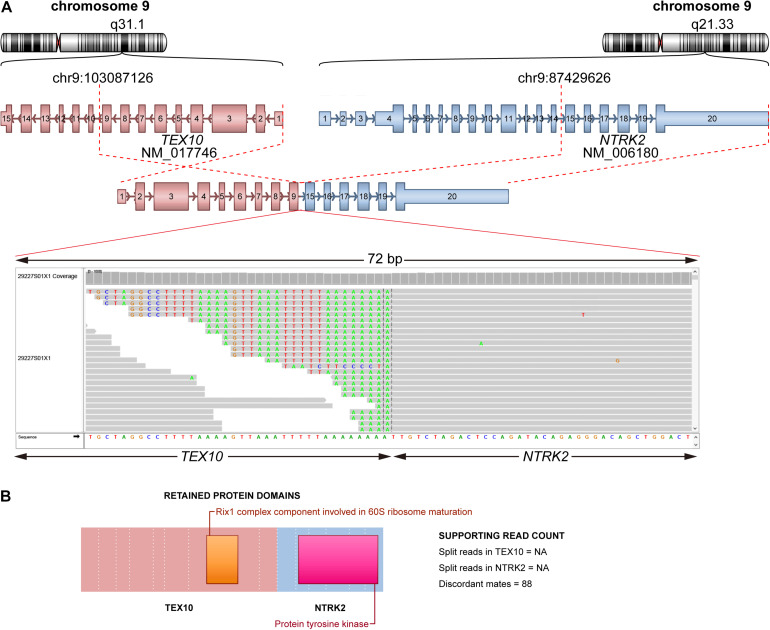
Schematic plot of fusion of *TEX10*-*NTRK2*. **(A)** Representation of fusion *TEX10*-*NTRK2*. Top: chromosome and cytoband where breakpoint located on for each partner gene. Middle: fusion form (*TEX10* exon 1–9 joined *NTRK2* exon 15–20). Bottom: reads spanning breakpoint using IGV. **(B)** Retained protein domains of each partner gene.

In total, there were 14 fusions in 14 patients detected in this study, among which 64.3% (9/14) of fusions were not found in the COSMIC database (Supplementary Table 4). Of note, one patient had *TEX10*-*NTRK2* fusion, which can benefit from larotrectinib and entrectinib (Cocco et al., 2018). This fusion was annotated as a join of exon 9 of *TEX10* to exon 15 of *NTRK2* (Figure 3A), and the tyrosine kinase domain of *NTRK2* was retained (Figure 3B). Thus, this fusion can produce a functional fusion transcript. We also found two patients with an *FGFR3-TACC3* fusion that could be a target for cancer therapy [29].

### Deregulated Cancer-Related Pathways in the Chinese Head and Neck Squamous Cell Carcinoma Cohort

**FIGURE 4:**
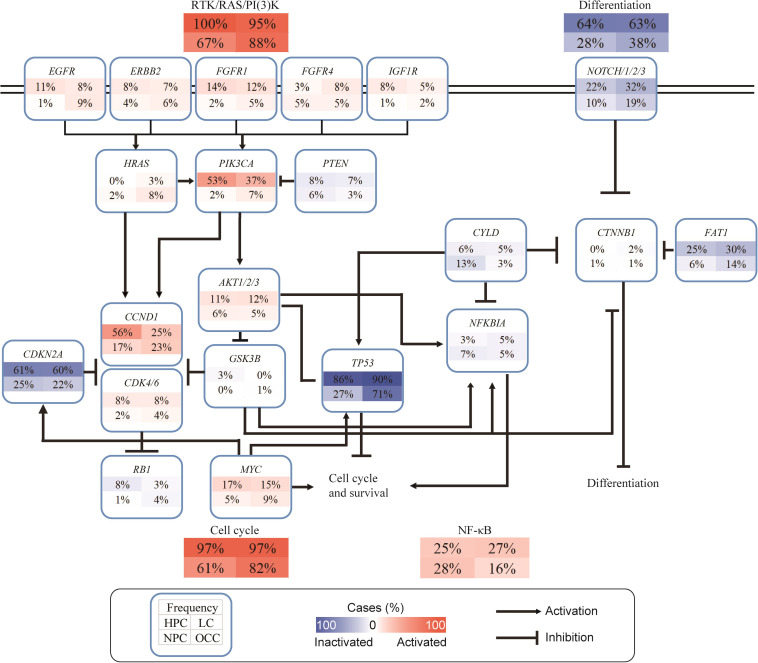
Deregulated cancer-related pathways in Chinese HNSCCs. Pathway alterations include SNVs, INDELs, and CNVs. Each number in box indicates percentage (%) of altered samples of given gene or pathway. Frequency of different anatomic sites is shown separately within subpanels. Activated and inactivated pathways or genes are based on predicted effects of alterations and/or pathway functions.

To further understand the function of the identified gene alterations in this Chinese cohort, SNVs/INDELs and CNVs were combined together to perform the pathway analysis, and a limited number of cancer-related pathways targeted by frequent genome alterations were frequently deregulated in HNSCC (Figure 4). Most of the HPC and LC samples had at least one gene alteration belonging to the receptor tyrosine kinase (RTK)/RAS/phosphatidylinositol-3-OH kinase (PI3K) pathway. The alteration frequency of the RTK/RAS/PI3K pathway in this Chinese cohort was much higher than that in the TCGA HNSCC dataset (Cancer Genome Atlas Network (CGAN), 2015). Among the RTKs, *FGFR1* alterations were the most frequent, followed by *EGFR* and *ERBB2* alterations. For the downstream of the RTK/RAS/PI3K pathway, *PIK3CA* had the highest alteration frequency. Interestingly, the alteration frequencies of *PIK3CA* varied greatly across different anatomic sites. More than half of the HPC samples had *PIK3CA* alterations, whereas only 2% NPC and 7% OCC samples altered in this gene. For the downstream cell cycle pathway, a total of 97% HPC, 97% LC, 61% NPC, and 82% OCC samples were altered. Among components of the cell cycle pathway, *TP53* alterations dominated, followed by *CDKN2A*. *CCND1* and *MYC* were the two most frequently altered oncogenes of this pathway. Further alterations of *NOTCH1* and *FAT1* linked functionally to β-catenin (*CTNNB1*) are also detected. As tumor-suppressor genes, the inactivation of *FAT1* and *NOTCH1* may converge to inhibit the Wnt/β-catenin signaling pathway, which is implicated in the deregulation of cell polarity and differentiation. Compared with the patients from the other anatomic sites, a higher alteration frequency in the nuclear factor kappa-light-chain-enhancer of activated B cells (NF-κB) signaling pathway was found for NPC patients, although the alteration frequencies in the other pathways were lower for them. *CYLD* alterations dominated in this pathway, followed by *NFKBIA* (Figure 4).

### Identification and Characterization of the Actionable Alterations

To identify and characterize the actionable alterations in this Chinese cohort, different kinds of genetic alterations types, including SNVs/INDELs, CNVs, fusions, and MSI (Supplementary Table 5) status, were combined and annotated using the OncoKB knowledge database (Chakravarty et al., 2017). OncoKB is a database containing information about the effects and treatment implications of specific cancer gene alterations, which is curated from guidelines from the Food and Drug Administration, National Comprehensive Cancer Network, or American Society of Clinical Oncology, ClinicalTrials.gov, and the scientific literature. According to the annotation results (Supplementary Table 6), 85.5% (271/317) of patients had at least one oncogenic mutation, and 44.5% (141/317) had at least one actionable alteration. The evidence levels of most actionable alterations were levels 3 and 4. We summarized all actionable alterations to 37 kinds of alterations based on gene symbol and variant type (Supplementary Table 7). *CDKN2A* deletion was the most frequent actionable alteration affecting 15.1% of total cases, followed by *CDKN2A* SNVs/INDEs covering 11.4% of all cases. There were 5% of patients with actionable alterations on *PIK3CA* SNVs/INDEs of level 3B. Of note, there were three patients with level 1 alteration, including one patient with *TEX10-NTRK2* fusion and two patients with MSI-H.

## Discussion

In this study, we delineated the genome alteration landscape of HNSCC in a Chinese cohort across different anatomic sites. Overall, the mutation loads and CNV frequency in NPC were lower than those in the other anatomic sites (Figures 1A, 2A), consistent with other studies (Lin et al., 2014; Zheng et al., 2016; Li et al., 2017). Thus, we confirmed the molecular characteristics of the Chinese population. Among the recurrently mutated genes, *TP53* was the most highly mutated gene across different anatomic sites. The mutation frequency of *TP53* in NPC was much lower than that in the other sites, indicating that the mechanisms of tumor tumorigenesis may be different between NPC and other HNSCC sites.

HPC and LC were found with higher CNV alteration frequency compared with NPC and OCC (Figure 2A). *PIK3CA* had the highest CNV discrepancy across the four anatomic sites (Figure 2B). *PIK3CA* is a well-known oncogene that can play an important role in cancer development. *PIK3CA* gains also recurrently occurred in TCGA HNSCC dataset (Cancer Genome Atlas Network (CGAN), 2015). However, *PIK3CA* gain was much lower in OCC in the current cohort than in TCGA OCC (1.8 vs. 15.1%; *P* = 3.4e-04, proportion test). The co-amplification of *FGF3*, *FGF4*, *FGF19*, and *CCND1* in HNSCC patients was most likely due to co-localization of the loci in the same chromosomal region (11q13) (Parish et al., 2015).

Most genes and pathways analyzed in NPC were relatively altered in a lower frequency; however, *CYLD* and NF-κB signaling pathways in NPC had a higher mutation frequency than the other sites (Figure 4). A previous study detected enrichment of genomic abnormalities of multiple negative regulators of the NF-κB pathway, including *CYLD*, *TRAF3*, *NFKBIA*, and *NLRC5* (Li et al., 2017). In this study, such observation confirmed that genes altered in the NF-κB signaling pathway had a higher mutation frequency in NPC than in the other sites. Activation of NF-κB is associated with a poorer prognosis in NPC (Zhang et al., 2011; Sun et al., 2012), supporting the rationale for exploiting NF-κB inhibition as a potential treatment strategy for NPC. Several studies have demonstrated that targeting the NF-κB pathway is a promising treatment strategy in a variety of cancers (Sunwoo et al., 2001; Lun et al., 2005; Zhang et al., 2015). Thus, it is rational to apply the NF-κB activation status to stratify NPC patients who may be more likely to benefit from treatment with NF-κB inhibitors. *CYLD* is a tumor suppressor gene encoding a cytoplasmic protein that functions as a deubiquitinating enzyme that deubiquitinates *TRAF2/5/6*, which is an agonist of the NF-κB signaling pathway (Sun, 2010). A previous study also reported that *CYLD* could regulate p53 DNA damage response by removing K48-linked ubiquitin chains from p53 (Fernandez-Majada et al., 2016). Interestingly, the mutation of *CYLD* and *TP53* was absolutely mutually exclusive in NPC patients, indicating different roles of *CYLD* and *TP53* in NPC oncogenesis.

Most HNSCC samples harbor potentially actionable mutations. Using genomic profiling, we discovered the potentially actionable mutation (either directly targeted or a pathway component of a directly targeted gene by an approved or investigational drug), which could be targeted with either an off-label or an investigational therapeutic available in a clinical trial. Nearly half of the HNSCC patients were identified with clinically actionable alterations in this cohort. Therefore, these patients may benefit from Food and Drug Administration-approved drugs or clinical trials. *TEX10-NTRK2* fusion was identified in an LC patient, and the patient may benefit from larotrectinib or entrectinib with level 1 evidence, and two MSI-H patients may benefit from Keytruda. Although the majority of alterations were used as biomarkers in clinical trials, patients with these alterations may also benefit from clinical trials.

The limitations of this study are worth mentioning. First, the observed differences across different anatomic sites need verification. Second, the genes significantly different between this Chinese cohort and TCGA dataset also need verification.

In conclusion, we performed targeted sequencing in 317 Chinese HNSCC patients. The molecular characteristics and differences were analyzed and compared across different anatomic sites. Actionable mutations were identified, and patients who may benefit from the actionable alterations from clinical treatment were also identified.

## Data Availability Statement

The datasets presented in this study can be found in online repositories. The names of the repository/repositories and accession number(s) can be found below: Figshare Database: https://figshare.com/projects/Genomic_landscape_of_head_and_neck_squamous_cell_carcinoma_across_different_anatomic_sites_in_Chinese_population/111842.

## Ethics Statement

The studies involving human participants were reviewed and approved by the ethics committee of the Third Affiliated Hospital of Kunming Medical University. The patients/participants provided their written informed consent to participate in this study.

## Author Contributions

SY, FL, HC, NX, and DZ: conception and design. SY and SZ: administrative support. YJ, XW, and QL: provision of study materials or patients. YJ, XW, HW, and QL: collection and assembly of data. YJ, XW, HW, BL, SY, and SZ: data analysis and interpretation. YJ, XW, HW, BL, QL, DZ, HC, NX, FL, SY, and SZ: manuscript writing and final approval of manuscript.

## Conflict of Interest

HW, BL, DZ, HC, NX, and FL were employed by company 3D Medicines Inc. The remaining authors declare that the research was conducted in the absence of any commercial or financial relationships that could be construed as a potential conflict of interest.

## Footnotes

1 https://broadinsti tute.github.io/picard/

## References

[B1] Boffetta P. Hecht S. Gray N. Gupta P. Straif K. (2008). Smokeless tobacco and cancer. *Lancet Oncol.* 9 667–675. 10.1016/S1470-2045(08)70173-6 18598931

[B2] Bray F. Ferlay J. Soerjomataram I. Siegel R. L. Torre L. A. Jemal A. (2018). Global cancer statistics 2018: GLOBOCAN estimates of incidence and mortality worldwide for 36 cancers in 185 countries. *CA Cancer J. Clin.* 68 394–424. 10.3322/caac.21492 30207593

[B3] Cancer Genome Atlas Network (CGAN). (2015). Comprehensive genomic characterization of head and neck squamous cell carcinomas. *Nature* 517 576–582. 10.1038/nature14129 25631445 PMC4311405

[B4] Chakravarty D. Gao J. Phillips S. M. Kundra R. Zhang H. Wang J. (2017). OncoKB: a Precision Oncology Knowledge Base. *JCO Precis. Oncol.* 2017:PO.17.00011. 10.1200/PO.17.00011 28890946 PMC5586540

[B5] Chaturvedi A. K. Engels E. A. Pfeiffer R. M. Hernandez B. Y. Xiao W. Kim E. (2011). Human papillomavirus and rising oropharyngeal cancer incidence in the United States. *J. Clin. Oncol.* 29 4294–4301. 10.1200/JCO.2011.36.4596 21969503 PMC3221528

[B6] Chen W. Zheng R. Baade P. D. Zhang S. Zeng H. Bray F. (2016). Cancer statistics in China, 2015. *CA Cancer J. Clin.* 66 115–132. 10.3322/caac.21338 26808342

[B7] Cocco E. Scaltriti M. Drilon A. (2018). NTRK fusion-positive cancers and TRK inhibitor therapy. *Nat. Rev. Clin. Oncol.* 15 731–747. 10.1038/s41571-018-0113-0 30333516 PMC6419506

[B8] Du E. Mazul A. L. Farquhar D. Brennan P. Anantharaman D. Abedi-Ardekani B. (2019). Long-term Survival in Head and Neck Cancer: impact of Site, Stage, Smoking, and Human Papillomavirus Status. *Laryngoscope* 129 2506–2513. 10.1002/lary.27807 30637762 PMC6907689

[B9] Fernandez-Majada V. Welz P. S. Ermolaeva M. A. Schell M. Adam A. Dietlein F. (2016). The tumour suppressor CYLD regulates the p53 DNA damage response. *Nat. Commun.* 7:12508. 10.1038/ncomms12508 27561390 PMC5007442

[B10] Gandini S. Botteri E. Iodice S. Boniol M. Lowenfels A. B. Maisonneuve P. (2008). Tobacco smoking and cancer: a meta-analysis. *Int. J. Cancer* 122 155–164. 10.1002/ijc.23033 17893872

[B11] Hashibe M. Boffetta P. Zaridze D. Shangina O. Szeszenia-Dabrowska N. Mates D. (2006). Evidence for an important role of alcohol- and aldehyde-metabolizing genes in cancers of the upper aerodigestive tract. *Cancer Epidemiol. Biomarkers Prev.* 15 696–703. 10.1158/1055-9965.EPI-05-0710 16614111

[B12] India Project Team of the International Cancer Genome Consortium (IPTICGC). (2013). Mutational landscape of gingivo-buccal oral squamous cell carcinoma reveals new recurrently-mutated genes and molecular subgroups. *Nat. Commun.* 4:2873. 10.1038/ncomms3873 24292195 PMC3863896

[B13] Izumchenko E. Sun K. Jones S. Brait M. Agrawal N. Koch W. (2015). Notch1 mutations are drivers of oral tumorigenesis. *Cancer Prev. Res.* 8 277–286. 10.1158/1940-6207.CAPR-14-0257 25406187 PMC4383685

[B14] Leemans C. R. Braakhuis B. J. Brakenhoff R. H. (2011). The molecular biology of head and neck cancer. *Nat. Rev. Cancer* 11 9–22. 10.1038/nrc2982 21160525

[B15] Li H. Durbin R. (2010). Fast and accurate long-read alignment with Burrows-Wheeler transform. *Bioinformatics* 26 589–595. 10.1093/bioinformatics/btp698 20080505 PMC2828108

[B16] Li S. Lee Y. C. Li Q. Chen C. J. Hsu W. L. Lou P. J. (2015). Oral lesions, chronic diseases and the risk of head and neck cancer. *Oral Oncol.* 51 1082–1087. 10.1016/j.oraloncology.2015.10.014 26526128

[B17] Li Y. Y. Chung G. T. Lui V. W. To K. F. Ma B. B. Chow C. (2017). Exome and genome sequencing of nasopharynx cancer identifies NF-kappaB pathway activating mutations. *Nat. Commun.* 8:14121. 10.1038/ncomms14121 28098136 PMC5253631

[B18] Lin D. C. Meng X. Hazawa M. Nagata Y. Varela A. M. Xu L. (2014). The genomic landscape of nasopharyngeal carcinoma. *Nat. Genet.* 46 866–871. 10.1038/ng.3006 24952746

[B19] Lun M. Zhang P. L. Siegelmann-Danieli N. Blasick T. M. Brown R. E. (2005). Intracellular inhibitory effects of Velcade correlate with morphoproteomic expression of phosphorylated-nuclear factor-kappaB and p53 in breast cancer cell lines. *Ann. Clin. Lab. Sci.* 35 15–24. 15830705

[B20] Ma J. Fu Y. Tu Y. Y. Liu Y. Tan Y. R. Ju W. T. (2018). Mutation allele frequency threshold does not affect prognostic analysis using next-generation sequencing in oral squamous cell carcinoma. *BMC Cancer* 18:758. 10.1186/s12885-018-4481-8 30041611 PMC6057048

[B21] Nakagaki T. Tamura M. Kobashi K. Koyama R. Fukushima H. Ohashi T. (2017). Profiling cancer-related gene mutations in oral squamous cell carcinoma from Japanese patients by targeted amplicon sequencing. *Oncotarget* 8 59113–59122. 10.18632/oncotarget.19262 28938622 PMC5601718

[B22] Niedobitek G. (2000). Epstein-Barr virus infection in the pathogenesis of nasopharyngeal carcinoma. *Mol. Pathol.* 53 248–254. 11091848 10.1136/mp.53.5.248 PMC1186977

[B23] Parish A. Schwaederle M. Daniels G. Piccioni D. Fanta P. Schwab R. (2015). Fibroblast growth factor family aberrations in cancers: clinical and molecular characteristics. *Cell Cycle* 14 2121–2128. 10.1080/15384101.2015.1041691 25950492 PMC4614941

[B24] Song X. Xia R. Li J. Long Z. Ren H. Chen W. (2014). Common and complex Notch1 mutations in Chinese oral squamous cell carcinoma. *Clin. Cancer Res.* 20 701–710. 10.1158/1078-0432.CCR-13-1050 24277457 PMC3946562

[B25] Stransky N. Egloff A. M. Tward A. D. Kostic A. D. Cibulskis K. Sivachenko A. (2011). The mutational landscape of head and neck squamous cell carcinoma. *Science* 333 1157–1160. 10.1126/science.1208130 21798893 PMC3415217

[B26] Su D. Zhang D. Chen K. Lu J. Wu J. Cao X. (2017). High performance of targeted next generation sequencing on variance detection in clinical tumor specimens in comparison with current conventional methods. *J. Exp. Clin. Cancer Res.* 36:121. 10.1186/s13046-017-0591-4 28882180 PMC5590190

[B27] Sun S. C. (2010). CYLD: a tumor suppressor deubiquitinase regulating NF-kappaB activation and diverse biological processes. *Cell Death Differ.* 17 25–34. 10.1038/cdd.2009.43 19373246 PMC5848464

[B28] Sun W. Guo M. M. Han P. Lin J. Z. Liang F. Y. Tan G. M. (2012). Id-1 and the p65 subunit of NF-kappaB promote migration of nasopharyngeal carcinoma cells and are correlated with poor prognosis. *Carcinogenesis* 33 810–817. 10.1093/carcin/bgs027 22301282

[B29] Sunwoo J. B. Chen Z. Dong G. Yeh N. Crowl Bancroft C. Sausville E. (2001). Novel proteasome inhibitor PS-341 inhibits activation of nuclear factor-kappa B, cell survival, tumor growth, and angiogenesis in squamous cell carcinoma. *Clin. Cancer Res.* 7 1419–1428. 11350913

[B30] Tao Y. Gross N. Fan X. Yang J. Teng M. Li X. (2018). Identification of novel enriched recurrent chimeric COL7A1-UCN2 in human laryngeal cancer samples using deep sequencing. *BMC Cancer* 18:248. 10.1186/s12885-018-4161-8 29499655 PMC5834868

[B31] Tu C. Zeng Z. Qi P. Li X. Guo C. Xiong F. (2018). Identification of genomic alterations in nasopharyngeal carcinoma and nasopharyngeal carcinoma-derived Epstein-Barr virus by whole-genome sequencing. *Carcinogenesis* 39 1517–1528. 10.1093/carcin/bgy108 30102338

[B32] Vermorken J. B. Mesia R. Rivera F. Remenar E. Kawecki A. Rottey S. (2008). Platinum-based chemotherapy plus cetuximab in head and neck cancer. *N. Engl. J. Med.* 359 1116–1127. 10.1056/NEJMoa0802656 18784101

[B33] Vermorken J. B. Trigo J. Hitt R. Koralewski P. Diaz-Rubio E. Rolland F. (2007). Open-label, uncontrolled, multicenter phase II study to evaluate the efficacy and toxicity of cetuximab as a single agent in patients with recurrent and/or metastatic squamous cell carcinoma of the head and neck who failed to respond to platinum-based therapy. *J. Clin. Oncol.* 25 2171–2177. 10.1200/JCO.2006.06.7447 17538161

[B34] Vogelstein B. Papadopoulos N. Velculescu V. E. Zhou S. Diaz L. A. Jr. Kinzler K. W. (2013). Cancer genome landscapes. *Science* 339 1546–1558. 10.1126/science.1235122 23539594 PMC3749880

[B35] Yoshizaki T. Kondo S. Endo K. Nakanishi Y. Aga M. Kobayashi E. (2018). Modulation of the tumor microenvironment by Epstein-Barr virus latent membrane protein 1 in nasopharyngeal carcinoma. *Cancer Sci.* 109 272–278. 10.1111/cas.13473 29247573 PMC5797826

[B36] Young L. S. Dawson C. W. (2014). Epstein-Barr virus and nasopharyngeal carcinoma. *Chin. J. Cancer* 33 581–590. 10.5732/cjc.014.10197 25418193 PMC4308653

[B37] Zhang L. Macisaac K. D. Zhou T. Huang P. Y. Xin C. Dobson J. R. (2017). Genomic Analysis of Nasopharyngeal Carcinoma Reveals TME-Based Subtypes. *Mol. Cancer Res.* 15 1722–1732. 28851814 10.1158/1541-7786.MCR-17-0134

[B38] Zhang X. A. Zhang S. Yin Q. Zhang J. (2015). Quercetin induces human colon cancer cells apoptosis by inhibiting the nuclear factor-kappa B Pathway. *Pharmacogn. Mag.* 11 404–409. 10.4103/0973-1296.153096 25829782 PMC4378141

[B39] Zhang Y. Chen Y. Yu J. Liu G. Huang Z. (2014). Integrated transcriptome analysis reveals miRNA-mRNA crosstalk in laryngeal squamous cell carcinoma. *Genomics* 104 249–256. 10.1016/j.ygeno.2014.06.004 24979738

[B40] Zhang Y. Lang J. Y. Liu L. Wang J. Feng G. Jiang Y. (2011). Association of nuclear factor kappaB expression with a poor outcome in nasopharyngeal carcinoma. *Med. Oncol.* 28 1338–1342. 10.1007/s12032-010-9571-7 20499210

[B41] Zheng H. Dai W. Cheung A. K. Ko J. M. Kan R. Wong B. W. (2016). Whole-exome sequencing identifies multiple loss-of-function mutations of NF-kappaB pathway regulators in nasopharyngeal carcinoma. *Proc. Natl. Acad. Sci. U. S. A.* 113 11283–11288. 10.1073/pnas.1607606113 27647909 PMC5056105

