# Supplementary figures and images for: Genomic Landscape of Head and Neck Squamous Cell Carcinoma Across Different Anatomic Sites in Chinese Population
Source: Front Genet. 2021 Jun 14;12:680699. doi: 10.3389/fgene.2021.680699 (PMC8236955; doi:10.3389/fgene.2021.680699)

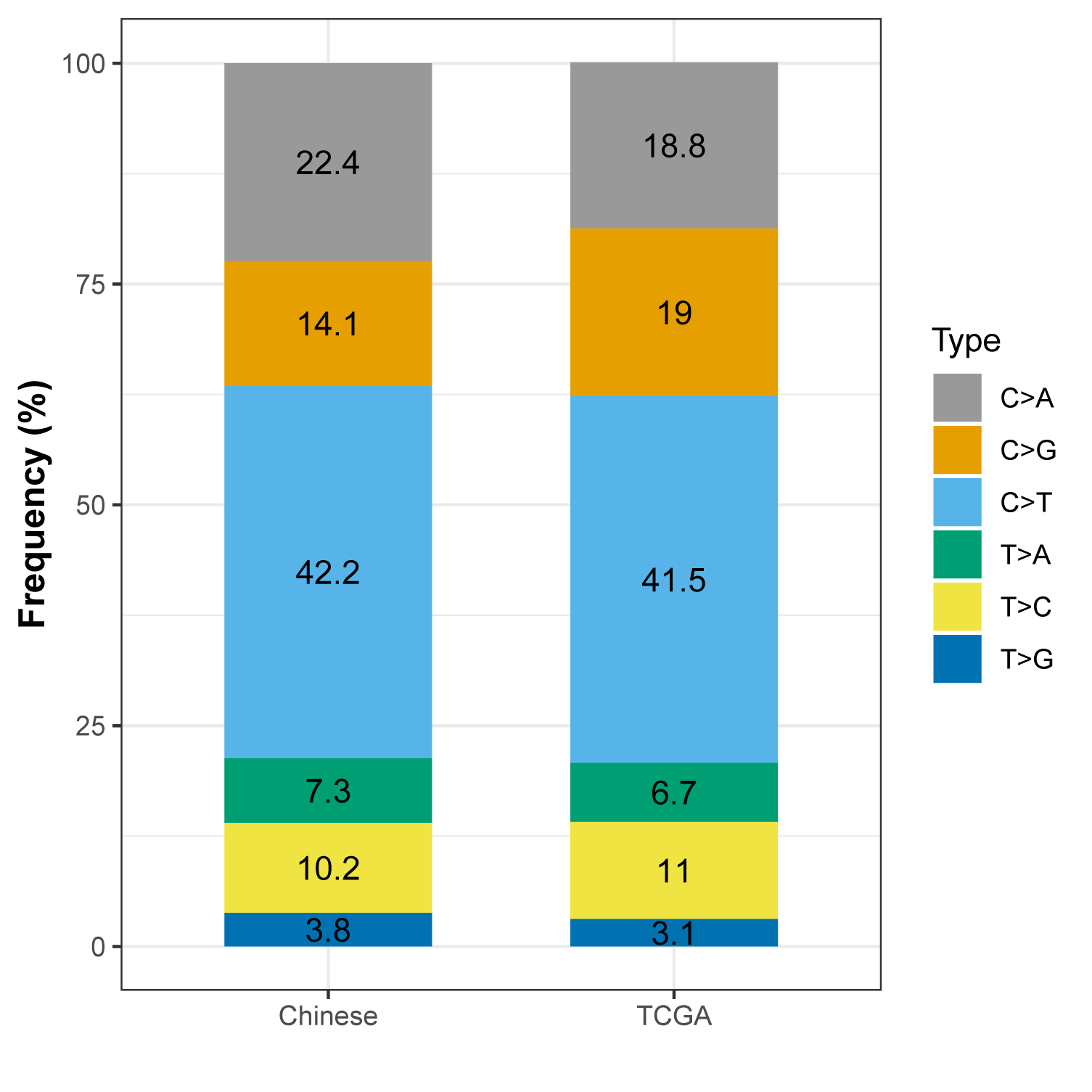

Supplement: Supplementary file 2 [file Image_1.TIF]

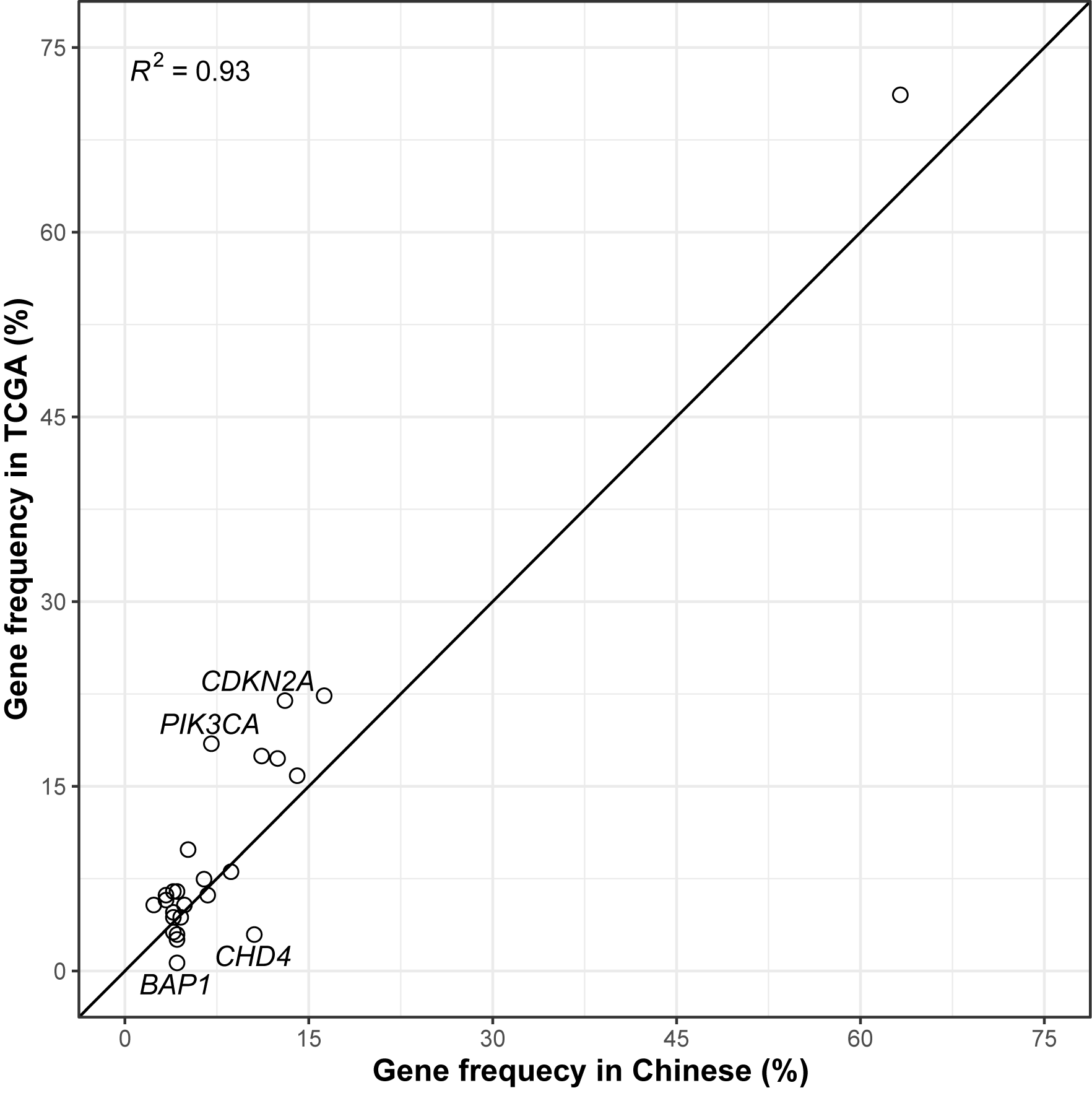

Supplement: Supplementary file 3 [file Image_2.TIF]

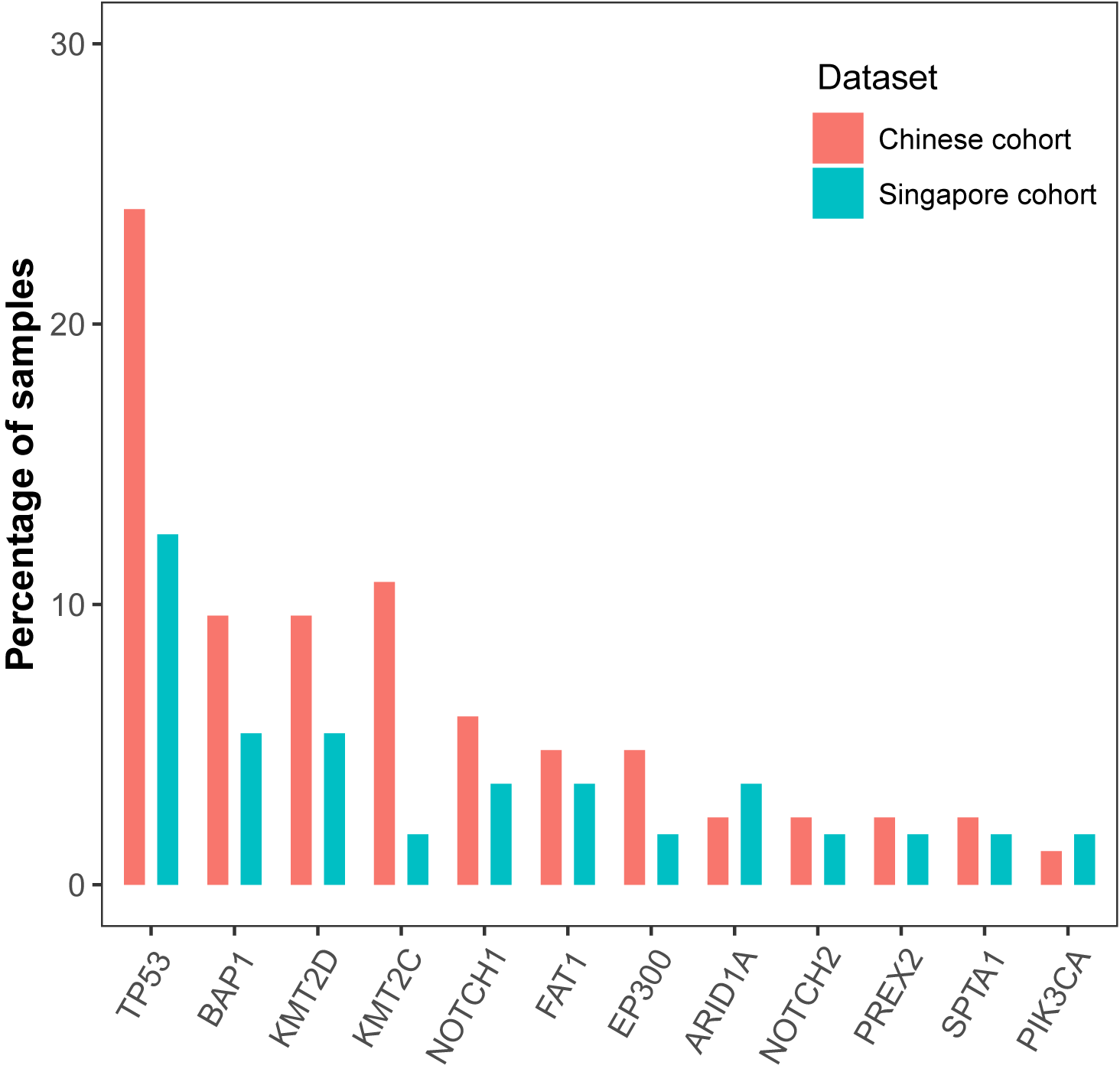

Supplement: Supplementary file 4 [file Image_3.TIF]

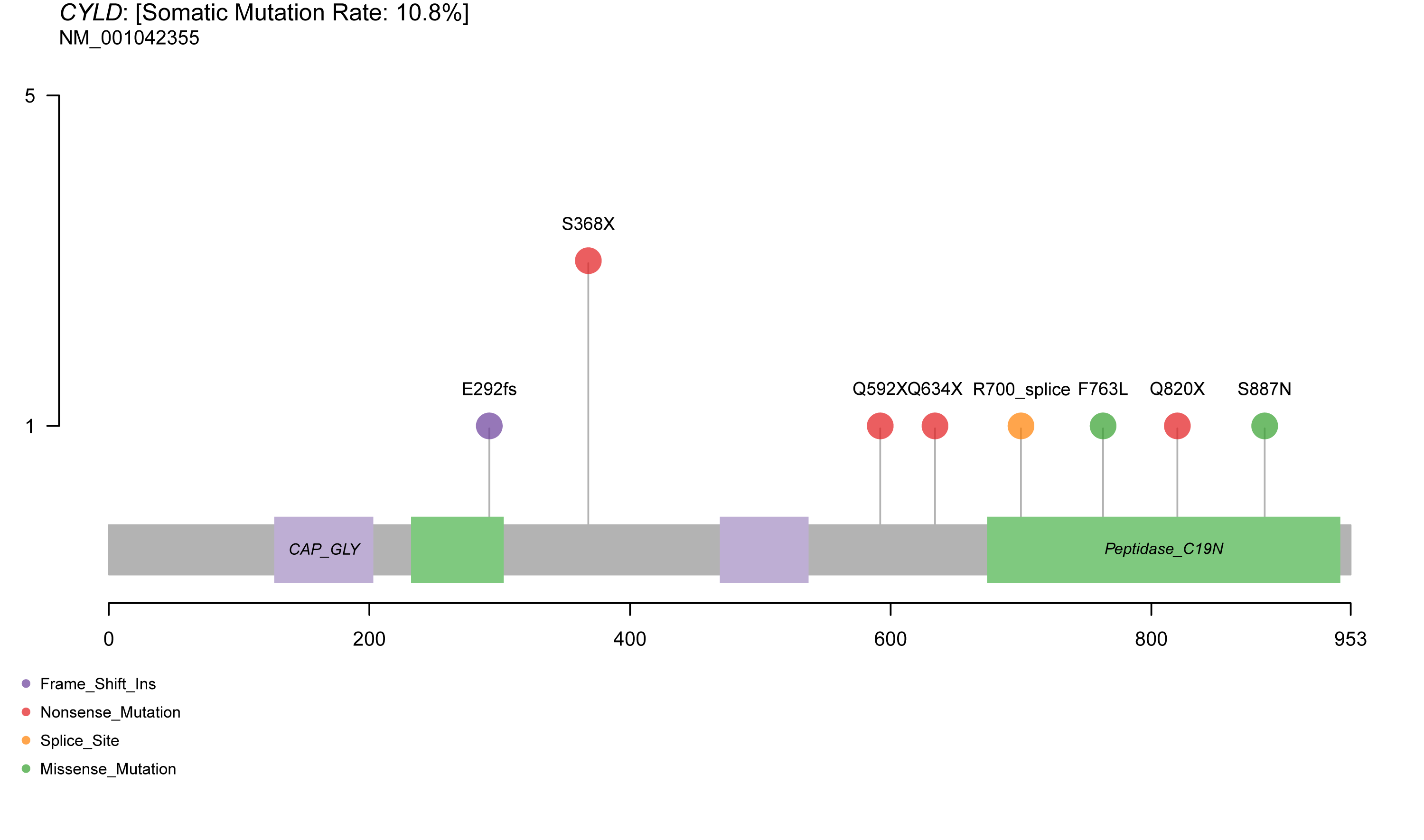

Supplement: Supplementary file 5 [file Image_4.TIF]

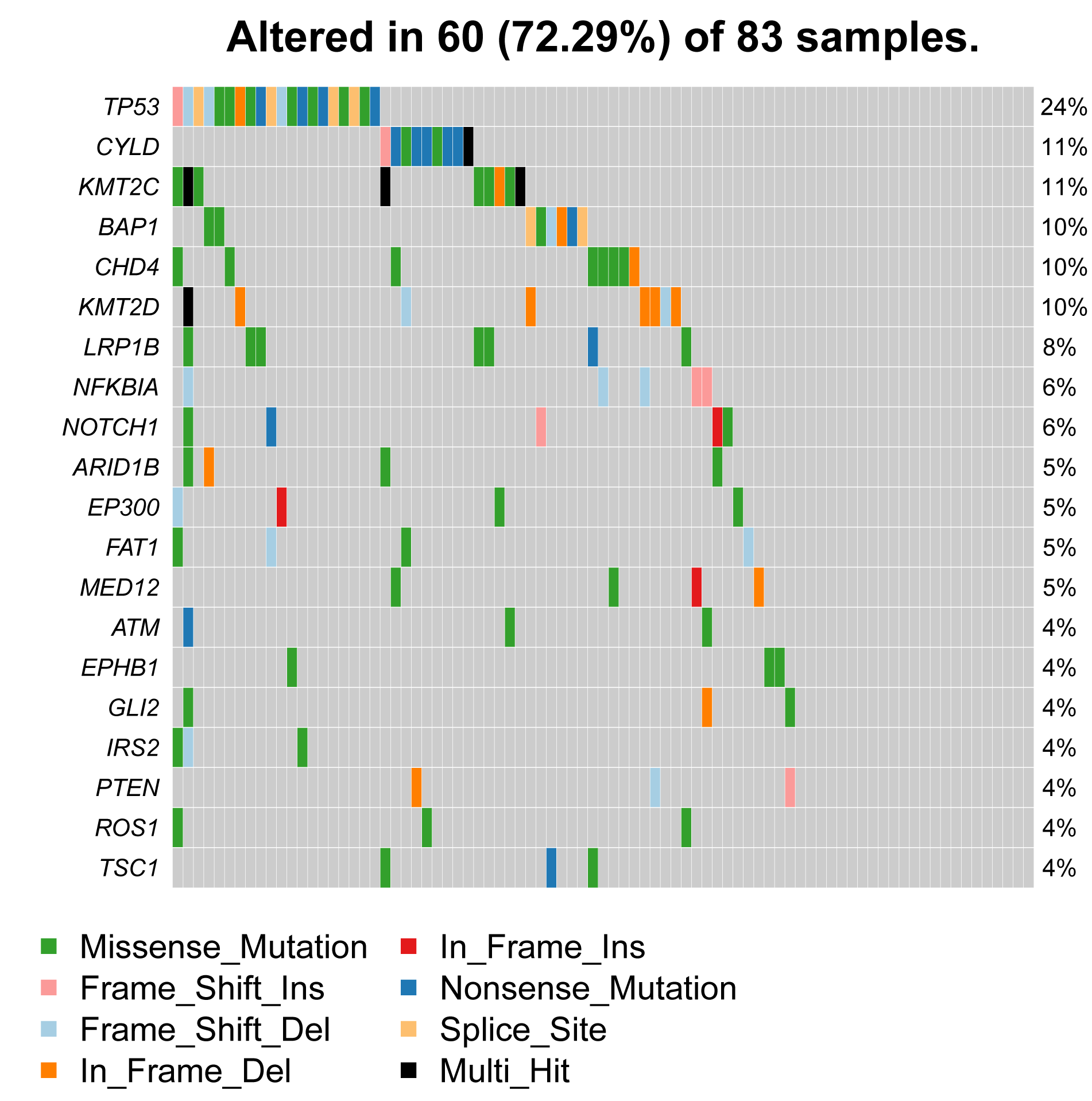

Supplement: Supplementary file 6 [file Image_5.TIF]
